# Nitrogen-Doped Graphene Quantum Dots Conjugated to Leucettinib-21 Rescue Differentiating Zebrafish Purkinje Cells by Inhibiting Dyrk1A Kinase

**DOI:** 10.1021/acsanm.6c00239

**Published:** 2026-04-20

**Authors:** Luiza Araújo Gusmão, Annemarie Metzke, Emmanuel Deau, Laurent Meijer, Antonio Claudio Tedesco, Reinhard W. Köster

**Affiliations:** 1 Division of Cellular and Molecular Neurobiology, Zoological Institute, 26527 Technische Universität Braunschweig, Spielmannstraße 7, Braunschweig 38106, Germany; 2 Department of Chemistry, Center of Nanotechnology and Tissue Engineering, Photobiology and Photomedicine Research Group, Faculty of Philosophy, Sciences and Letters of Ribeirão Preto, University of São Paulo, São Paulo 05508-220,Brazil; 3 Perharidy Research Center, Perha Pharmaceuticals, Roscoff 29680, France

**Keywords:** zebrafish, graphene, quantum dots, cerebellum, Purkinje cell, Dyrk1A, Leucettinib

## Abstract

A major challenge in treating neurological diseases is the transport of compounds across the blood–brain barrier. Herein, we report the synthesis and characterization of nitrogen-doped graphene quantum dots (GQDs) that exhibit high tolerance in zebrafish larvae at high concentrations. In contrast to classical semiconductor quantum dots, vascular microinjection of these fluorescent carbon-based nanomaterials results in rapid tissue distribution and efficient neuronal internalization within the brain, highlighting their potential as nanocarriers for central nervous system delivery. Vascular microinjections of these quantum dots conjugated with the high-affinity Dyrk1A kinase inhibitor Leucettinib-21 (LCTB21) at nanomolar concentrations rescued cell-autonomous dendrite deficiencies in cerebellar Purkinje cells overexpressing human Dyrk1a. LCTB21 concentrations were significantly lower than those of the inhibitor alone. Dyrk1A activity is responsible for neurological defects in Down syndrome and acts as a priming kinase for Alzheimer’s disease-associated proteins Tau and APP. Thus, efficient nanodelivery of Dyrk1A inhibitors across the blood–brain barrier improves therapeutic options while minimizing the treatment dose and potential side effects.

## Graphical abstract

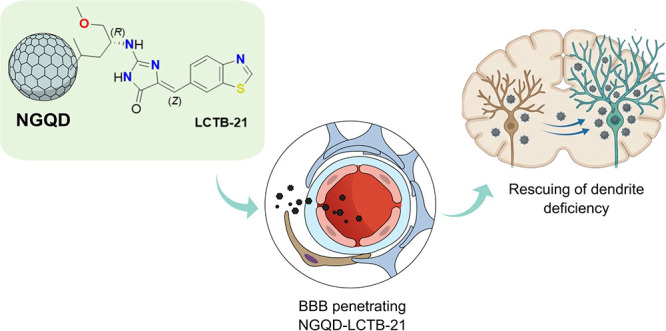

## Introduction

Quantum dots (QDs) are nanoparticles with discrete fluorescence emissions. Initially, they were synthesized from semiconductor materials such as GaAs, CdSe, or CdTe and shielded by coats or enclosed in lipid micelles.^1,2^ QDs have significant advantages over organic fluorescent dyes for cellular imaging, including low cytotoxicity and high resistance to photobleaching. This enhanced photostability enables prolonged fluorescence and repeated imaging.^3^ Similar small particles from carbon, called carbon dots (CDs), when nonfluorescent, and carbon quantum dots (CQDs), when fluorescent, have demonstrated superior biocompatibility. Surface functional groups improve their solubility and enable functionalization.^4^ Enclosing graphene sheets in CDs produces graphene quantum dots (GQDs) with excellent luminescent properties. Doping GQDs with nitrogen improves their fluorescence and enhances their *in vivo* tolerance. Microwave-assisted synthesis allows for the cost-efficient preparation of nitrogen-doped GQDs (NGQDs) using a carbon source such as glucose or citric acid and a nitrogen source such as ethylenediamine or urea.^5^

These GQDs were rapidly tested for their therapeutic potential alone or in combination with drugs, yielding promising results. For example, GQDs have been shown to inhibit the aggregation of Aβ-peptides in the formation of amyloid structures observed in Alzheimer’s disease (AD), and conjugating GQDs to tramiprosate further enhances the inhibition of aggregation.^6,7^ GQDs have potential as antidiabetic, antimicrobial, or anticancer agents, and as nanocarriers for substance delivery inside cells.^5^ The most challenging applications target neurological diseases, because therapeutic compounds must effectively cross the blood-brain barrier (BBB). In this study, GQDs successfully delivered neuroprotective peptides into the brains of AD mouse models to alleviate AD symptoms.^8^ However, the fluorescence properties of GQDs cannot be fully harnessed in mice without invasive procedures.

In contrast, zebrafish larvae develop in an aqueous environment, facilitating the application of quantum dots (QDs). Since the first use of QDs in zebrafish,^9^ their larvae have become a prominent model for characterizing the cytotoxicity, bioavailability, and therapeutic properties of nanoparticles. These studies have revealed the tremendous potential of GQDs as therapeutic agents,^10–12^ but also the potential health risks associated with GQDs, which can induce ER stress, neurological defects, or alter gene expression.^13–15^ Because of the large variety of GQDs, their safety or toxicity cannot be generalized; they need to be tested individually depending on the concentration applied, with NGQDs displaying low toxicity and superior photoluminescence properties.^5,16^ To avoid cytotoxic effects during therapy, NGQDs should be noncovalently conjugated with high-affinity pharmacological compounds active in the nanomolar range.

A prime example is Leucettinib-21 (LCTB21), which acts as a high-affinity inhibitor of the highly conserved dual-specificity tyrosine phosphorylation-regulated kinase 1A (Dyrk1A) by competitively binding to the ATP-binding site of this kinase.^17,18^ The human *dyrk1a* gene is located on chromosome 21 in the Down syndrome (DS) critical region; hence, this kinase is overexpressed in individuals with DS-causing trisomy 21.^19^ Dyrk1A overexpression causes developmental defects in neurons and leads to neurological deficits in individuals with DS.^20^ In addition to affecting cell cycle exit, Dyrk1A regulates synaptogenesis in developing neurons.^21,22^ Furthermore, Dyrk1A functions as a priming kinase for the AD-causing Amyloid Precursor Protein and Tau.^23,24^ Genome-wide association studies have revealed that Dyrk1A is a risk factor for Parkinson’s disease (PD).^25^ Therefore, Dyrk1a inhibitor development is crucial, among which LCTB21 demonstrates high activity at the nanomolar level and superior drug candidate properties for DS, AD and PD.^17,18,26^ However, the ability of this inhibitor to rescue Dyrk1A hyperactivity phenotypes at the individual neuronal level remains unclear.

Overexpression of human Dyrk1A in zebrafish Purkinje cells (PCs) results in cerebellar differentiation defects that affect PC synaptogenesis, which can be rescued by Dyrk1a inhibitors.^27^ Here, we investigated the cell-autonomy of phenotypes in single PCs overexpressing Dyrk1A. Furthermore, we examined the ability of LCTB21 to rescue neuronal defects when delivered into the vasculature of the animals. Importantly, we investigated whether NGQDs could potentiate the efficacy of LCTB21 by facilitating its entry into the central nervous system (CNS). These studies are significant because DS, AD, and PD are prevalent neurological and neurodegenerative disorders in humans, for which only symptomatic treatments are currently available. The effective delivery of inhibitors targeting hyperactive pathogenic factors at the lowest possible dose is necessary to minimize adverse effects, prevent cellular habituation, and enable flexible dosing strategies.

## Experimental Section

### Synthesis of NGQDs

Citric acid monohydrate (0.3 g, C1909, Sigma-Aldrich, St. Louis, MO, USA) and urea (0.9 g, 2317.3, Carl Roth GmbH, Karlsruhe, Germany) were dissolved in 10 mL of deionized water. Microwave-assisted carbonization (1050 W, 2.5 min) was followed by dissolving the synthesized NGQDs in 30 mL of deionized water, filtration (0.22 μm), and drying at 60 °C (48 h) to obtain a homogeneous powder and to determine the yield. NGQDs were dissolved in water, stored at 4 °C, and protected from light.

### Synthesis of Leucettinib-21 and Iso-Leucettinib-21

Leucettinib-21 (LCTB21, MW: 358.46g/mol) and iso-Leucettinib-21 (iso-LCTB21) were synthesized as previously described.^18^ They were stored as powders or as 10 mM stock solutions in DMSO and diluted immediately before use.

### Characterization and Loading of NGQDs

Spectrophotometric analyses were employed to characterize and quantify the concentrations of NGQD through absorption measurements in the UV–Vis region (DS5 Dual Beam, Edinburgh Instruments Ltd., UK) and fluorescence spectroscopy (Fluorolog-3 Horiba Jobin Yvon, Oberursel, Germany). NGQD’s structure were characterized using X-ray diffraction (Bruker-AXS, model D2 PHASER, Bruker, Billerica, MA, USA). The surface functional groups were analyzed using Fourier-Transform Infrared (FTIR) spectroscopy (Spectrum Two educational System, PerkinElmer, Waltham, MA, USA) and Raman spectroscopy (XPlora Plus, Horiba Scientific, Irvine, CA, USA). The hydrodynamic size and polydispersity index were measured by dynamic light scattering with a He–Ne laser operating at 633 nm, a scattering angle of 173°, at 25.0 °C ± 0.1 °C. The zeta potentials were obtained by investigating the electrophoretic mobility of the samples in suspension (Zetasizer Nano ZS ZEN3600, Malvern PCS Instruments, Malvern, UK).

The brain penetrance properties of LCTB21 and isoLCTB21 were predicted *in silico* using the SwissADME program (http://www.swissadme.ch/) and displayed as a boiled-egg graph.^28,29^

To generate NGQD/LCTB21 conjugates for cardiac ventricle injections, NGQDs (100 μg/mL) and LCTB21 or iso-LCTB21 (MW: 358.46 g/mol each) were diluted in HPLC water (A511.1, Carl Roth GmbH, Karlsruhe Germany) to their respective concentrations (50 nM, 500 nM) and incubated at room temperature for 16 h on a rotator (300 rpm). Subsequently, the mixtures were sterile-filtered through a 0.22 μm cellulose acetate membrane (Ahlstrom Reliaprep 760516, Finland) and stored at 4 °C.

### Blood–Brain Barrier Permeability (PAMPA-BBB Assay)

Passive permeability was evaluated using a PAMPA-BBB-adapted model. A 0.4 μm PET membrane insert (6-well format) (Millipore, Massachusetts, USA) was impregnated with a lipid solution in n-dodecane to form a biomimetic lipid barrier. The donor compartment (1.0 mL) contained the test compound in PBS (pH 7.4), and the acceptor compartment (2.0 mL) contained the PBS. Fluorescein, methylene blue, and rhodamine B were used as permeability controls to validate membrane integrity. Aliquots were removed every 5 min for 1 h from the acceptor compartment to calculate the apparent permeation rate (P_app_). The Papp parameter was calculated from the rate of compound appearance in the acceptor chamber, normalized to membrane area and initial donor concentration.^30^ All experiments were performed in triplicate.

### Plasmid Construction

All expression vectors were generated in pBluescript SKII (Stratagene, USA) or pCS2+ vector backbones^31^ using restriction enzyme-mediated cloning strategies (New England Biolabs, Ipswich, MA, USA), followed by plasmid DNA preparation (Macherey Nagel GmbH, Düren, Germany) and sequence analysis (Eurofins, Luxembourg).

pCS-mem-mScarlet: The red fluorescent mScarlet protein was fused at its N-terminus with the membrane-targeting peptide MLCCIRRTKPVEKNEEADQEGST of the zebrafish GAP43 protein.

cDNA of human Dyrk1A kinase (isoform 2, NP_001334650.1, kindly provided by Walter Becker) fused with a dual HA tag sequence at its N-terminus was used to construct the PC-specific hDyrk1A expression construct. The kinase-inactivating mutation (K179R, corresponding to K188R in isoform1 ^32,33^ was introduced using a nested PCR approach. hDyrk1A encoding cDNAs were inserted as *EcoR*I/*Xba*I fragments into bidirectional PC-specific expression vectors coexpressing a membrane-targeted EGFP fluorescent protein (same targeting peptide from GAP43 as shown for membrane-targeted mScarlet above) as a reporter.^34^

Further details regarding the cloning procedures are available upon request.

### Cell Culture

Zebrafish Pac2 cells were cultured and transfected, as previously described.^35^ Shortly, 50.000 cells were seeded into the wells of a 6-well plate and incubated in Leibovitz’s L-15 medium (Thermo Fischer Scientific, Waltham, MA, USA) supplemented with 15% Fetal Bovine Serum (Capricorn Scientific, Ebersdorfergrund, Germany), and 1% l-Glutamine/1% Penicillin/Streptomycin (Thermo Fischer Scientific). 0.5 μL of plasmid DNA (1 μg/μL) was transfected using 50 μL JetOptimus transfection reagent (Sartorius AG, Göttingen, Germany) according to the manufacturer’s instructions.

For primary cell culture, zebrafish larvae of the transgenic strain Tg­(*Xla*.Tubb:DsRed) were injected at 3dpf with 22 nL of NGQDs (16.6 mg/mL) into the cardiac ventricle. After 1 h, the larvae were used for primary neuronal cell culture, as previously described.^36^ In short, zebrafish larvae were enzymatically (Neural Tissue Dissociation Kit (T), #130–093–231, Miltenyi Biotec, Bergisch Gladbach, Germany) and physically (gentleMACS Dissociator #130–093–235, Miltenyi Biotec) dissociated, strained (40 μm cell strainer #542040, Greiner Bio-one, Kremsmünster, Austria) and plated onto coverslips coated with poly-l-lysine (0.5 mg/mL, #P9155, Sigma-Aldrich, St. Louis, MO, USA).

### Zebrafish Husbandry

Zebrafish were maintained and bred according to standard procedures and legal regulations (EU Directive 2010_63)^37^ by the local authorities, the animal welfare representative of the Braunschweig University of Technology, and the Lower Saxony State Office of Consumer Protection and Food Safety (LAVES, Oldenburg, Germany; Az. §4 (02.05) TSchB TU BS). Experiments are reported according to the ARRIVE guidelines. No selection criteria were used to differentiate between male and female zebrafish larvae. Embryos and larvae were raised in 30% Danieau rearing medium (100% Danieau: 58 mM NaCl, 0.7 mM KCl, 0.4 mM MgSO_4_, 0.6 mM Ca­(NO_3_)_2_, and 5 mM HEPES [4-(2-hydroxyethyl)-1-piperazineethanesulfonic acid] pH 7.2) at 28 °C. The stable transgenic reporter lines used in this study included Tg­(Xla.Tubb:DsRed, previously NBT-dsRED),^38^ Tg­(flk1:mCherry)^y206^ ^39^ and Tg­(ca8-E1B:FMATagRFP)^bz4^.^34^ Euthanasia of larvae was performed according to legal regulations using an overdose of Tricaine (0.25 mg/mL, Merck KGaA, Darmstadt, Germany) for 5 min until no heartbeat was detectable anymore.

Biosafety studies of NGQDs were performed by raising fertilized zebrafish eggs in 6-well plates (n= 15 per well) until 5dpf in 4 mL 30%Danieau supplemented with NGQDs at varying concentrations. Larvae were monitored daily using a transmitted light stereomicroscope (Leica Microsystems, Wetzlar, Germany), and their survival rates, hatching rates, and potential malformations were documented.

### Microinjection and Transgenesis

Zebrafish embryos were injected at the one-cell stage with plasmid DNA (25 ng/μL, about 1.5 nL injection volume) supplemented with 25 ng/μL mRNA encoding Tol2 transposase and 0.05% PhenolRed (Sigma-Aldrich, St. Louis, MO, USA). At 10hpf, the medium was supplemented with 0.003% phenylthiourea (PTU, Sigma-Aldrich, St. Louis, MO, USA) to prevent pigmentation. Larvae expressing the injected construct in a suitable number of cells were selected at 4dpf for further microscopic analysis.

For brain ventricle injections, 5dpf zebrafish larvae were anesthetized with 0.02% Tricaine dissolved in 30%Danieau, mounted with the dorsal hindbrain facing upward in 1.5% agarose/30% Danieau and overlaid with 30%Danieau/Tricaine (Merck KGaA, Darmstadt, Germany). The injection glass capillaries were loaded with NGQDs at a concentration of 16.6 mg/mL. Using a micromanipulator, the injection needle was inserted into the IVth ventricle in the hindbrain, and approximately 11 nL of NGQD solution was released twice (22 nL in total) into the cerebrospinal fluid, where NGQDs were quickly distributed throughout the central canal of the CNS. Microangiographic injections into zebrafish larvae at 3dpf or 5dpf were performed analogously, but with mounting of zebrafish larvae on their lateral side with the beating heart facing upward. The injection needle was placed inside the cardiac ventricle, where approximately 11 nL of NGQD solution was released twice (22 nL in total) into the bloodstream, which quickly distributed fluorescent nanoparticles throughout the vasculature.^40^

### Microscopy and Image Analysis

For vital membrane labeling, 4dpf zebrafish larvae were incubated in red fluorescent Bodipy (2.5 ng/μL diluted with 30% Danieau from 2.5 μg/μL stock in DMSO, BODIPY 558/568C_12_, D3835, Thermo Fisher Scientific) overnight and washed in 30% Danieau before NGQD brain ventricle injections.^41^

Larvae were anesthetized in 0.02% Tricaine (Merck KGaA, Darmstadt, Germany) and subsequently embedded in 1.5% ultralow melting agarose dissolved in 30% Danieau/PTU in a custom-made glass-bottom imaging chamber.^41^ Images were recorded with a confocal microscope (SP8 Leica Microsystems) using a 40 × Apochromat water immersion objective (NA 1.2) and subsequently processed using LAS X (Leica Microsystems, Wetzlar, G) and Fiji, ImageJ2 ^42^ software.

A program to remove fluorescent skin from intensity projections (FLAREZZ) can be found in the Github repository Koester-ZFish-Lab.

Sholl analysis: Images were exported to Fiji and converted into maximum intensity projections to outline the dendritic tree and remove the soma using Plugin NeuronJ software. Automated Sholl analysis determined dendrite contacts with concentric circles (15 μm) centered on the soma.^43^

### Statistical Analysis

Prism v8.0.2 (Graphpad Software Inc., San Diego, CA, USA) was used for the statistical analysis. Sum of intersection: The data were not normally distributed, according to the Shapiro-Wilk test, therefore nonparametric multiple comparison analysis (Benjamini, Krieger, and Yekutieli) was applied. Intersections per distance: Similarly, the Shapiro-Wilk test showed non-normal data, therefore, Friedman’s test was used for comparisons.

## Results

### Synthesis and Spectral Characterization of NGQDs

NGQDs were synthesized from citric acid and urea for zebrafish applications, as described in the literature.^44,45^ Urea and citric acid were dissolved in water at a 10.5:1 molar ratio and subjected to a microwave-assisted thermal treatment for carbonization. The solid was redispersed in water, filtered, and dried to obtain a dark-brown powder (Figure 1A). A dilution of 1.45 mg/mL of this powder resulted in a yellow solution (Figure 1B), which, when illuminated with UV light (365 nm), produced a bright green emission (Figure 1C). This phenomenon highlights the fluorescence properties of the NGQDs, as evidenced by the Stokes shift. The absorption spectrum indicated a maximum peak at 410 nm, linked to an n-π* transition due to nitrogen doping (Figure 1D). In addition, three absorption bands at 251, 272, and 330 nm were attributed to π-π* transitions.^46^ Because nitrogen doping increases structural defects and facilitates the conjugation of the π-system, it improves the electronic properties and increases the fluorescence quantum yield of NGQDs.^47^ This N-doping contributes to the band shift of graphene, typically from the UV to the visible region of the spectrum (red-shift).^16,48^ The 3D fluorescence emission spectra (Figure 1E) showed that an excitation range of 350–500 nm resulted in a narrow and well-defined emission centered at 525 nm, corresponding to the green region of the visible spectrum. Maximum fluorescence was obtained at an excitation wavelength of 410 nm. The NGQD absorption and emission maxima (410/525 nm) fit well with the commercially available light sources and chromophores commonly used in biological experiments.

**Figure 1:**
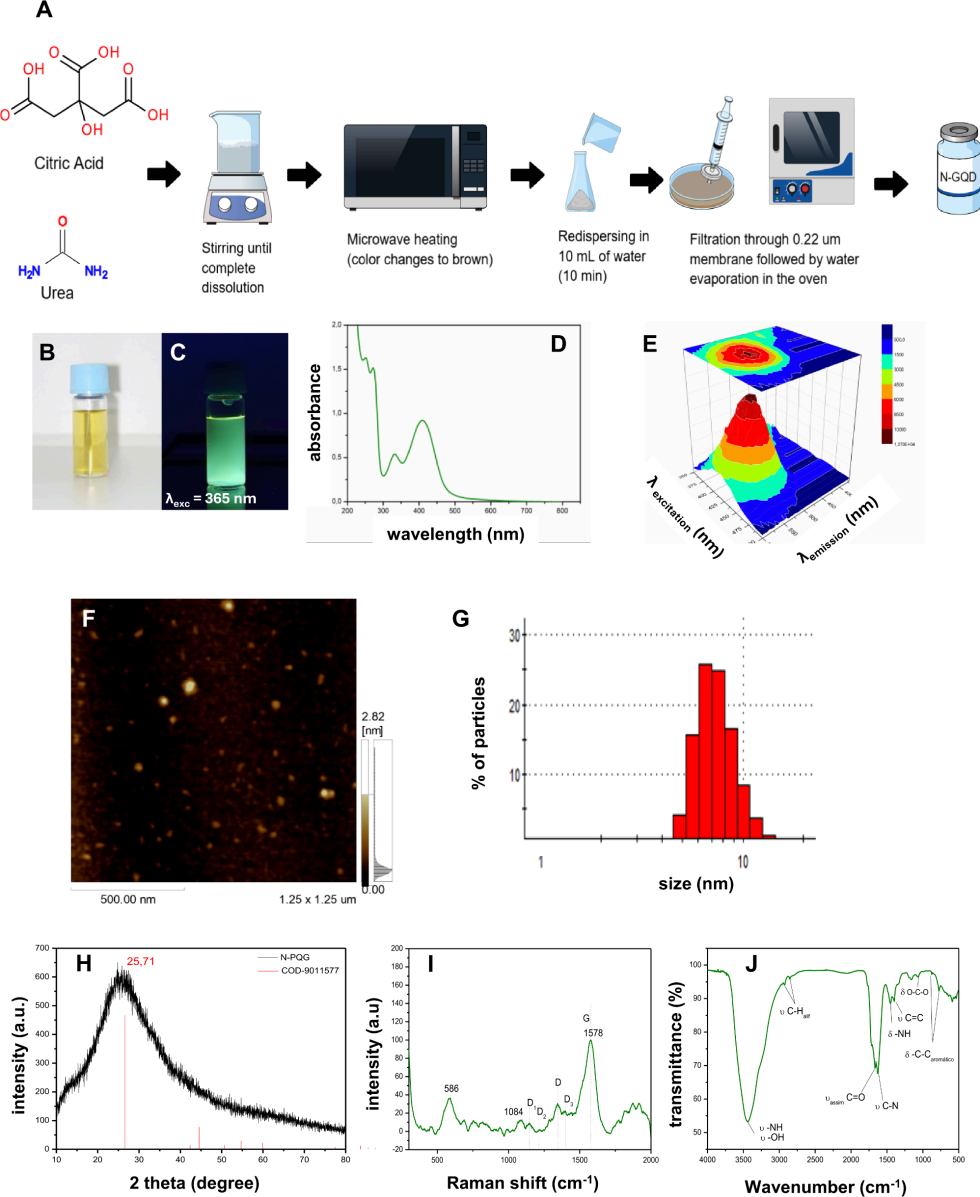
Synthesis, structural and optical analyses of NGQDs. (A) Schematic representation of the synthesis process used to obtain nitrogen-doped graphene quantum dots (NGQDs). Citric acid was used as the primary carbon source, and urea was used as the nitrogen source. The material was dissolved in water and heated in a microwave until carbonization. The resulting material was redispersed in water, filtered through a 0.22 μm membrane, and dried to determine the exact concentration. The obtained NGQDs were stored in aqueous solution at 4 °C. (B) NGQD solution under ambient light, and (C) under ultraviolet light (365 nm), showing strong green emission. (D) UV–vis absorption spectrum of NGQDs in an aqueous solution (1.45 mg/mL). (E) 3D fluorescence emission spectrum of NGQDs in aqueous solution; excitation at different wavelengths results in emissions centered in the green region (525 nm). (F) Atomic force microscopy (AFM) images of NGQDs deposited on mica substrate. (G) Size distribution histogram of NGQDs in water using dynamic light scattering (DLS). (H) X-ray diffraction (XRD) pattern of the NGQDs indexed with the corresponding crystallographic file in red (COD 9011577). (I) Raman scattering spectrum of NGQDs with band assignments characteristic of carbon- and graphene-based materials. (J) Fourier-transform infrared (FTIR) spectrum of NGQDs in KBr pellets, with the respective vibrational band assignments indicating surface bonds.

### Size Determination Classifies NGQDs as Nanoparticles

The sizes of the fluorescent NGQDs were evaluated to identify them as QDs. AFM images showed a maximum height of 7.6 nm and a width of up to 50 nm, with 47% of the nanoparticles measuring up to 20 nm (Figure 1F). Colloidal QD structures typically have quasi-spherical shapes with comparable height and width. However, the larger height of the NGQDs suggests that they may have formed agglomerates during preparation. Moreover, the tip convolution effect of AFM imaging can distort height measurements, affecting the accuracy of the sample size.^49^ Dynamic light scattering (DLS) was used to analyze the hydrodynamic diameter of the NGQDs (Figure 1G), and a size range comparable to that of the AFM results was observed. The Gaussian curve was centered around sizes near 7 nm, confirming the AFM height measurements, whereas the vast majority of NGQDs were distributed in a size range of 4–11 nm (Figure 1G).

### Structural Organization Reveals N-Doped Graphene in NGQDs

The X-ray diffractogram indicated peaks at 26.56° for the NGQDs, which was close to the (002) plane of graphite/graphene (Figure 1H), which is typically observed between 26.4° and 26.6°, according to the XRD standard (COD 9011577) and the literature. This peak indicates the partial stacking of carbon sheets with a hexagonal structure. The width of the peak suggests low crystallinity and the presence of structural defects, which are common in graphene-based materials such as doped and functionalized QDs and reduced graphene oxide.^50,51^

Raman scattering analysis of NGQDs featured two distinct bands at D1 (1146 cm^–1^), D2 (1215 cm^–1^) and D3 (1398 cm^–1^) that revealed sp2-sp3 bonds between carbons, corresponding to the COOH/C–OH and C = O/C–O groups, respectively.^52–54^ The D-band in the Raman spectra indicates local disorder and defects concentrated at the graphene edges. These structural defects and low crystallinity, resulting from the irregular arrangement of graphene introduced by oxygen atoms and functional groups, were confirmed by the width of the observed XRD peak and were characteristic of N-doped graphene^51,54,55^ (Figure 1I). The G band at 1578 cm^–1^ is related to doubly degenerate phonon modes (longitudinal optical (LO) and transverse optical (TO), which correspond to the E_2g_ symmetry of sp2 carbon bonds.^54^ These findings confirmed the identity of the fluorescent nanoparticles as graphene-containing QDs. The FTIR spectrum shows a broad band at 3435 cm^–1^, indicating O–H and N–H bond stretching vibrations (Figure 1J). Additional bands observed at 2928 and 2852 cm^–1^ further indicate the presence of associated N–H bonds. C = O vibrational bands were assigned at 1665 cm^–1^, and C = C bond vibrations appeared between 1631 and 1622 cm^–1^. The band at 1450 cm^–1^ corresponds to N–H angular stretching, while the bands at 1398 and 1336 cm^–1^ indicate C–N stretching in aromatic compounds. Peaks at 1159 and 1073 cm^–1^ were assigned to the C–O vibrations of aromatic ethers.^52^ These findings are consistent with graphene structures, while amine vibrational stretching confirmed the N-doping efficiency and hydrophilicity of the NGQDs owing to hydrogen bonding.

### NGQDs Are Negatively Charged and Highly Stable in Aqueous Solution

The stability of NGQDs in aqueous media was assessed to determine their potential as fluorescent nanoparticles in biological systems. The zeta potential, which indicates the surface charge of the NGQDs, was measured over 60 days and remained stable at −23.5 ± 0.6 mV, with only a 2.6% variation, confirming their high stability in solution (Figure S1). The negative zeta potential was due to the carboxylic and carbonyl groups on the NGQD surface, which were enhanced by resonance in the graphene structure. This is in accordance with the FTIR findings, indicating high stability and solubility in water, and the potential for functionalization. These traits make NGQDs promising candidates for biological applications, such as fluorescent contrast and drug delivery agents.

### NGQDs Permeate Biological Membrane Models

To investigate the behavior of the synthesized NGQDs in an environment reminiscent of cellular membranes, similar to the BBB, a parallel artificial membrane permeation assay (PAMPA) was performed. This assay simulates the permeation of materials through a lipid bilayer. PAMPA is commonly employed in the preliminary evaluation of potential diagnostic or therapeutic candidates because of its cost-effectiveness, robustness, and high reproducibility, to predict passive diffusion of compounds or materials across lipid membranes.^56^ A 400 nm PET membrane was coated with a lipid layer composed of cholesterol, DPPC (1,2-dipalmitoyl-*sn*-glycero-3-phosphocholine), and phosphatidylcholine, which mimics the lipid composition of the BBB.^57^ Because this method was adapted, the laboratory-produced membranes were validated using dodecane as a solvent. Three fluorescent compounds with different polarity characteristics were selected, and the obtained values were aligned with those reported in the literature: fluorescein (1.24 × 10^–5^ cm/s), rhodamine B (6.82 × 10^–6^ cm/s), and methylene blue (9.94 × 10^–6^ cm/s), confirming the validity of the method.^58^

In the same assay, the NGQDs displayed an apparent permeability coefficient of 1.41 × 10^–4^ cm/s, which was significantly higher than that of the fluorescent probes used as controls. This may be attributed to the ultrasmall size of the nanomaterial, which favors interactions with lipid bilayers and facilitates diffusion across biomimetic membranes. Of note, it has been reported that decreasing the size of graphene dotes increases their permeation rate.^59^

It is important to consider that the PAMPA model was originally developed to evaluate the passive permeability of small drug-like molecules across artificial lipid membranes. Therefore, when applied to nanomaterials, the assay should be interpreted primarily as a preliminary screening of their ability to interact with and diffuse through lipid environments, rather than as a complete predictor of biological barrier transport. Nevertheless, the high apparent permeability observed for NGQDs suggests an efficient interaction between these particles and lipid environments.^60^ Combined with their rapid cellular internalization observed *in vitro* and intrinsic fluorescence properties, these NGQDs likely present favorable characteristics for bioimaging applications that warrant testing *in vivo.*

### NGQDs Are Well-Tolerated and Do Not Exert Any Obvious Cytotoxicity

For testing nanoparticle properties, zebrafish larvae are popular due to their small size, optical transparency, and easy compound administration in a suitable aquatic environment.^9,12,61^ Zebrafish embryos were incubated in NGQDs starting shortly after fertilization until 5dpf, when zebrafish began to become self-sufficient organisms actively searching for food. Varying NGQD concentrations up to 4 mg/mL were used, and the embryo survival was monitored daily. At all concentrations, NGQDs showed no cytotoxic or detrimental effects, resulting in survival rates near 100% (n = 15 embryos tested per concentration, three replicates each), and the treated larvae did not display any obvious malformations (Figure 2A,C). In addition, successful hatching from the chorion was observed using NGQD concentrations from 2 to 200 μg/mL, and in all these assays, the hatching rates of embryos reached 100% on day 3 of embryonic development (Figure 2B). These findings suggest that NGQDs have outstanding biocompatibility. Kang et al. showed that a concentration of 1.5 mg/mL resulted in over 80% embryo survival rate, with malformation levels almost identical to the control group, regardless of whether the carbon dots were introduced by microinjection or immersion.^62^ Based on the above *in vitro* and *in vivo* toxicological studies, GQD generally have low toxicity and high biocompatibility. Furthermore, the literature reports that GQDs produced by different synthesis methods exhibit varying toxicity profiles, and their effects on different cell types and species also differ.^63,64^

**Figure 2:**
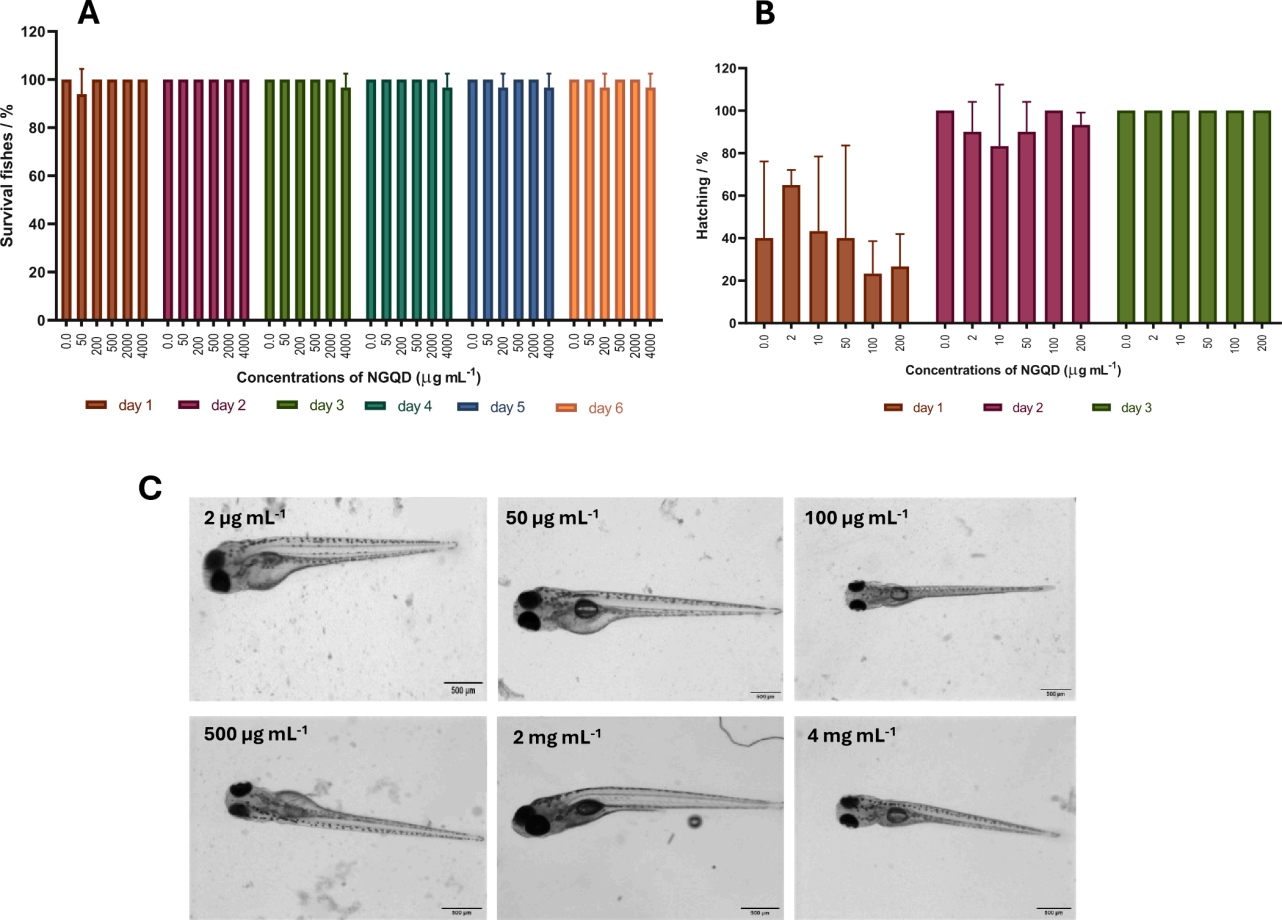
Biosafety assessment of NGQDs using zebrafish embryos. (A) Survival rates of zebrafish larvae incubated continuously over 5 days in increasing concentrations up to 4 mg/mL NGQDs dissolved in 30%Danieau. (B) Hatching rates of zebrafish larvae incubated continuously over 3 days in increasing concentrations from 2 μg/mL to 200 μg/mL of NGQDs dissolved in 30%Danieau. (C) Images of 5dpf old zebrafish larvae incubated continuously with increasing concentrations of NGQDs over 5 days. Images were recorded using a stereomicroscope (scale bar: 500 μm).

### Internalization of NGQDs by Zebrafish Cells in the CNS

Cultured zebrafish Pac2 fibroblasts were transfected with pCS-mem-mScarlet,^35^ a red fluorescent protein targeted to the cytoplasmic membrane, and incubated with NGQDs (2 μg/μL) for 6 h. Confocal microscopy revealed green fluorescent NGQD labeling of cellular membranes and vesicle-like structures confined to red fluorescent fibroblasts (Figure 3A), suggesting NGQD accumulation in lipophilic membranes and endocytic vesicles.

**Figure 3:**
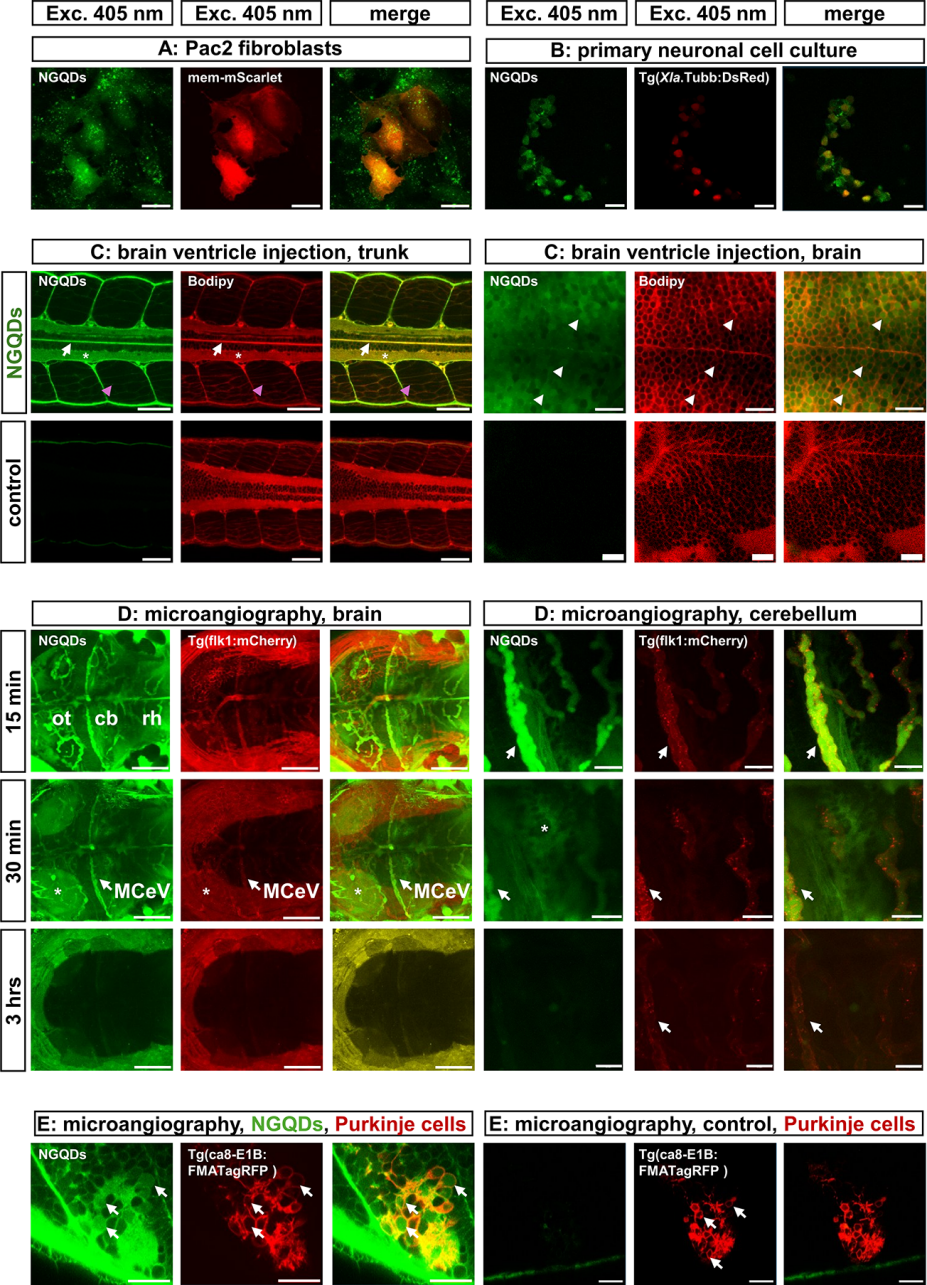
NGQDs accumulate in lipophilic cellular structures, cross the blood-brain barrier, and are internalized by cells of the CNS. (A) mem-mScarlet-expressing zebrafish Pac2 fibroblasts were incubated four 6 h with NGQD (2 μg/μL) (scale bar: 20 μm). (B) Cultured red fluorescent primary neurons obtained from Tg­(*Xla*.Tubb:DsRed) larvae after brain ventricle injection of NGQD (16.6 mg/mL) (scale bar: 10 μm). (C) Section (dorsal view, anterior to the left) of confocal microscopy recordings from 5dpf zebrafish larvae after NGQD injection into the brain ventricle: red fluorescence was derived from incubation in the lipophilic dye BODIPY 558/568C_12_. Images from NGQD-injected larvae are displayed in the upper row, while the lower row shows images from noninjected controls: green fluorescent NGQDs can be observed in the central canal of the neural tube (white arrow), in the neuropil (white asterisk), inside neural cells (white arrowheads), and in the membranes of somitic myocytes (pink arrowhead) (left panel: trunk, scale bar: 50 μm, right panel: brain, scale bar: 20 μm). (D) Maximum intensity projections (dorsal view, anterior to the left) from confocal microscopy recordings of the midbrain hindbrain area (left panel) or cerebellar tissue (right panel) of a 5dpf old Tg­(flk1:mCherry)^y206^ larva at different time points after microangiography using NGQDs (16.6 mg/mL). The middle cerebral vein MCeV is indicated by white arrows. Thirty min after microangiography, NGQD-derived green fluorescence was observed in the neuropil of the optic tectum (left panel, white asterisk, scale bar: 100 μm) and the cerebellum (right panel, white asterisk, scale bar: 20 μm). (E) Optical section (dorsal view, anterior to the left) from confocal microscopy recordings of the cerebellum of 5dpf old Tg­(ca8-E1B:FMATagRFP)^bz4^ larvae containing red fluorescent cerebellar Purkinje cells (white arrows) 1 h after NGQD microangiography. NGQD-treated larvae (left panel, scale bar: 20 μm) displayed NGQD-derived green fluorescence in the membranes and somata of cerebellar PCs, in contrast to a noninjected control larvae (right panel, scale bar: 20 μm). Abbreviations: cb cerebellum, MCeV middle cerebellar vein, NGQD nitrogen-doped graphene quantum dots, ot optic tectum, rh rhombencephalon.

Next, NGQDs were injected into the heart ventricle of 3dpf old zebrafish larvae of the transgenic strain Tg­(*Xla*.Tubb:DsRed) with pan-neuronal red fluorescent labeling of neurons.^38^ After 1 h, the larvae were used for primary neuronal cell culture.^36^ After 1 day, the cultured cells were fixed and characterized using confocal microscopy. The dsRed fluorescent protein-expressing neurons exhibited NGQD-derived green fluorescence (Figure 3B), indicating that NGQDs were internalized by zebrafish neurons *in vivo* and retained in these cells for at least 24 h.

To determine the NGQD distribution in the brain, zebrafish larvae stained with BODIPY 558/568C_12_ were mounted after brain ventricle injections at 5dpf and stacks of images were recorded using confocal microscopy. While red fluorescent Bodipy outlined cellular membranes in all larvae, only NGQD-injected larvae displayed green fluorescence, in contrast to noninjected control specimens (Figure 3C). In the trunks of NGQD-injected larvae (Figure 3C left panel), intense green fluorescence was observed throughout the neural tube cavity (white arrows) and inside the neuropil (white asterisk), marked by the lipophilic dye BODIPY. Interestingly, the membranes of somitic myocytes also displayed green fluorescence (pink arrowheads), suggesting that NGQDs enter the tissues outside the brain, likely by passing into the vasculature. In the brains of the NGQD-injected larvae (Figure 3C right panel) higher magnification revealed NGQD-derived green fluorescence not only in the Bodipy-stained red fluorescent membranes and neuropil, but also inside individual cells (white arrowheads), further supporting the observation from cell culture studies that NGQDs were internalized by cells of the CNS. Notably, the administration of these nanoparticles directly into the vasculature or CNS did not result in any obvious phenotypic changes, confirming the pronounced biotolerance of NGQDs.

### NGQDs Cross the Blood-Brain Barrier (BBB)

NGQD injections into the hindbrain ventricle of 5dpf zebrafish larvae resulted in fluorescent labeling of trunk myocytes, suggesting that NGQDs can cross the BBB (Figure 3C). Therefore, we microinjected NGQDs (22 nL of 16.6 mg/mL) dissolved in water into the cardiac ventricle of 5dpf transgenic Tg­(flk1:mCherry)^y206^ larvae, which contain red fluorescent blood vessels,^39^ and immediately analyzed the injected larvae using confocal microscopy. This showed that blood flow quickly distributed NGQDs throughout the larval vasculature, including the brain blood vessels. However, 15 min after injection, both the brain vasculature and the surrounding neural tissue displayed green NGQD-derived fluorescence (Figure 3D left panel). Thirty minutes after microangiography, blood vessel fluorescence decreased, whereas neural tissue fluorescence, particularly in neuropil-containing regions such as the optic tectum (white asterisks), increased. After approximately 3 h, NGQD-derived fluorescence was undetectable in both the vasculature and neural tissue, suggesting that the nanoparticles were cleared from the tissues or diluted in the surrounding tissues. The time-course of NGQD distribution became more evident when confocal microscopy analysis was focused at higher magnification on the area of the middle cerebral vein (MCeV)^65^ running along the dorsal part of the midbrain hindbrain boundary (Figure 3D right panel**,** white arrow). NGQDs labeled blood vessels 15 min after microangiography, with green fluorescence spreading into the cerebellum (white asterisk) by 30 min and disappearing after 3 h. These results confirm that NGQDs rapidly exit the vasculature, distribute in the surrounding tissues, and cross the BBB to enter the brain. In contrast, semiconductor QDs remain inside endothelial vessels and are removed by the lymphatic system.^9,40^

To further demonstrate that specific neuronal cell types can be supplied with NGQDs, microangiography was performed with 5dpf old larvae of the transgenic strain Tg­(ca8-E1B:FMATagRFP)^bz4,^ in which cerebellar PCs are marked by red fluorescence in their membranes.^34^ At this age, the BBB in zebrafish is established and functional, excluding molecules from the brain, except for the circumventricular organ.^66,67^ Optical sectioning using confocal microscopy 1 h after microangiography revealed that red fluorescence in the membranes of PCs colocalized with NGQD-derived green fluorescence (Figure 3E left panel, white arrows), which could not be observed in the PCs of control specimens without NGQD microangiography (Figure 3E, right panel, white arrows). In addition to the fluorescent membranes, NGQD fluorescence was observed inside the PC somata (white arrows). These observations show that NGQDs can quickly pass through the BBB, distribute throughout the CNS tissue, and are internalized by neurons in the brain. Currently, we cannot determine whether NGQDs are metabolized by cells or released via the lymphatic system. Nevertheless, NGQDs do not accumulate in cells, where increasing intracellular concentrations could become harmful over time, but instead, they possess a moderate turnover rate. This is important for materials carrying pharmacological compounds, which we tested for a novel high-affinity inhibitor of Dyrk1A kinase, LCTB21.

### Hyperactivity of the Human Kinase Dyrk1A Results in Cell-Autonomous Reduction of Dendrite Formation in Zebrafish Cerebellar PCs

We recently showed that the expression of human kinase Dyrk1A in zebrafish cerebellar PCs, in which *dyrk1a* is endogenously expressed, results in compromised differentiation of these neurons and a dramatic decrease in the synaptic density of PCs in adult zebrafish.^27^ In zebrafish, Dyrk1A activity needs to be well regulated, because knockdown or knockout of the *dykr1a* homologues results in impaired social behavior and compromised cerebral vascular development, whereas *dyrk1a* overexpression leads to primordial germ cell defects.^68–70^ *dyrk1a* homologues are expressed in various zebrafish brain tissues and are strongly expressed in the cerebellum, including in PCs.^27^ Population-wide overexpression of Dyrk1A in PCs resulted in morphological changes, likely caused by PC compaction and a reduction in synapse density in the adult cerebellum.

To reveal the morphological consequences of human Dyrk1A hyperactivity on the differentiation of PCs in a cell-autonomous manner, we established bicistronic expression vectors in which human Dyrk1A and a membrane-targeted enhanced green fluorescent protein (mem-EGFP) were coexpressed specifically in PCs mediated by four copies of the PC-specific *cpce* regulatory element.^34^ As a control, a “kinase dead” variant of human Dyrk1A (hDyrk1A-KD) containing a point mutation (K179R), which inactivates the catalytic activity of the kinase domain, was used^32,33^ (Figure 4A), as well as a vector expressing EGFP alone and lacking the *dyrk1a* cDNA (empty vector control). Fertilized zebrafish eggs were coinjected with these constructs and mRNA encoding Tol2 transposase to promote single-copy integration of the respective transgene cassettes flanked by Tol2-recognition sites.^71^ At 5dpf, zebrafish larvae with individual green-fluorescent PCs were selected for confocal microscopy.

**Figure 4:**
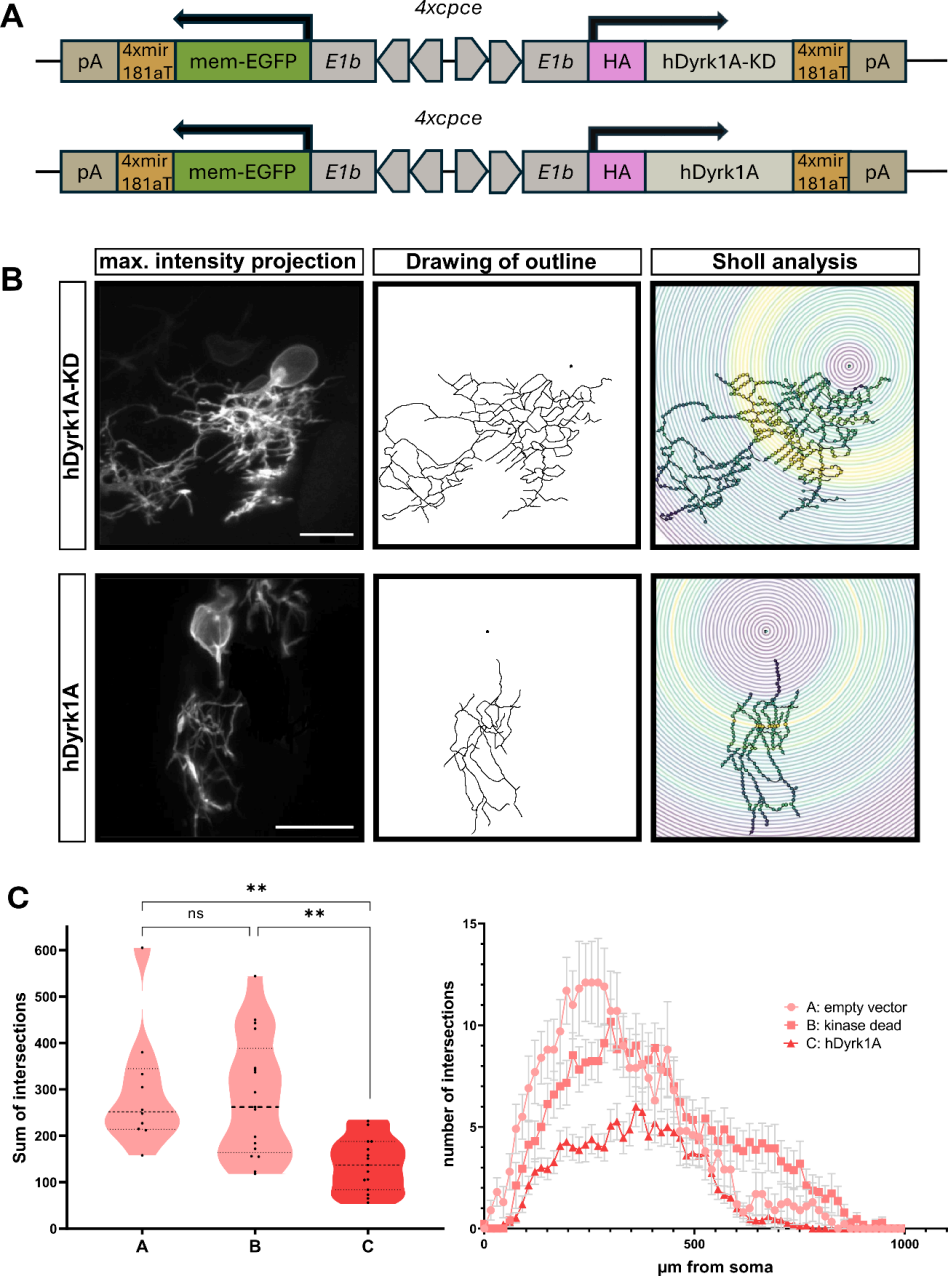
Overexpression of human Dyrk1A in zebrafish PCs results in reduced dendrite complexity. (A) Schematic drawings (not to scale) of PC-specific expression vectors mediating the expression of a membrane-targeted EGFP reporter, together with either Dyrk1A or catalytically inactive Dyrk1A-KD as control. (B) Individual cerebellar PCs recorded by confocal microscopy at 4dpf are displayed as maximum projections (left panel, scale bar: 10 μm), followed by digital reconstruction of their outline (middle panel), which was used for Sholl analysis (right panel) of dendrite intersections with concentric circles at a distance of 15 μm centered on the PC soma. (C) Results from the Sholl analysis either displayed as the total number of dendrite intersections (left graph, empty vector vs kinase dead *p* = 0.4013, empty vector vs hDyrk1A *p* < 0.0015, kinase dead vs hDyrk1A *p* < 0.0015, (A empty mem-EGFP only *n* = 10, B kinase dead *n* = 17, C hDyrk1A *n* = 15) or the average number of dendrite intersections in relation to the distance to the PC soma (right graph, empty vector vs kinase dead *p* > 0.9999, empty vector vs hDyrk1A *p* < 0.0001, kinase dead vs hDyrk1A *p* < 0.0001). Abbreviations: cpce *ca8* promoter derived PC specific enhancer element, E1b minimal basal promoter derived from adenovirus, EGFP enhanced Green Fluorescent Protein, HA hemagglutinin sequence tag, pA SV40 polyadenylation sequence.

These images revealed that the expression of human Dyrk1A in individual PCs resulted in a significant shortage of dendritic fields and a reduction in dendrite complexity as a cell-autonomous effect. This phenotype is dependent on the kinase activity of Dyrk1A, because the expression of a kinase-inactive Dyrk1A mutant did not affect PC dendrite morphology. PCs with reduced dendritic trees require less space, which explains PC-layer compaction in zebrafish with Dyrk1A overexpression. Furthermore, fewer and shorter dendritic branches provide less space for synapses, which explains the decrease in synaptic density in PCs with elevated Dyrk1A expression. This reduction in PC dendrite complexity is reminiscent of the observations in DS models in mice and AD-affected humans.^72,73^

For quantification, the recorded image stacks of individual transgene-expressing PCs were converted into maximum-intensity projections (Figure 4B, left panel), followed by tracing the outline of the dendritic tree (Figure 4B, middle panel). With these two-dimensional dendrite representations, Sholl analysis was performed (Figure 4B, right panel). This analysis determines the number of intersections of dendritic structures with concentric circles centered on the soma of the analyzed neuron.^43^ The number of intersections provides a means of determining the complexity of the dendritic tree concerning its distance from the cell soma.

With a total number of 293.90 (±120.53, n = 10 PCs analyzed) intersections in only EGFP-expressing PCs (empty vector control) and 283.06 (±125.98, n = 17) intersections of PCs expressing the catalytically inactive hDyrk1A (hDyrk1A-KD), no significant differences in the complexity of the individual dendritic trees were observed (Figure 4C, left graph). This indicates that the overexpression of catalytically inactive hDyrk1A does not affect the morphology of PC dendrites. This was further supported when the number of dendritic intersections was plotted against the distance from the soma. Both graphs were not significantly different and displayed a maximum of dendrite intersections at approximately 250 μm away from the PC soma and a total dendritic field length of approximately 850 μm (Figure 4C, right graph).

In contrast, when a catalytically active hDyrk1a kinase was expressed in individual PCs, the total number of dendrite intersections of 137.4 (±54.42, n = 15) in the Sholl analysis was significantly reduced compared to that in both controls (Figure 4C, left graph). A visual impression of stunted dendrite morphology in PCs with hDyrk1A hyperactivity (Figure 4B, lower panels, comparing the upper and lower images) was further corroborated by the plot of dendrite intersections against their distance from the soma (Figure 4C, right graph). PCs overexpressing hDyrk1A showed peaks of dendrite intersections that were shifted distally by approximately 100 μm to approximately 350 μm. However, this peak amounted to only approximately five dendrite intersections on average, compared to approximately 12 dendrite intersections in both types of control PCs at their intersection maxima. In addition, this graph revealed that hDyrk1A-expressing PCs displayed shortened dendritic trees, which, on average, terminated at approximately 650 μm from the soma and were approximately 200 μm shorter than control PCs. These results confirm previous findings that hDyrk1A overexpression impairs the maturation of differentiating cerebellar PCs in zebrafish^27^ and show that compromised dendrite outgrowth and branching are dependent on the kinase activity of the expressed hDyrk1A in a cell-autonomous manner.

### The hDyrk1A Inhibitor Leucettinib-21 Interacts with NGQDs

Pharmacological therapeutic approaches for neurological diseases using small molecules struggle to achieve effective concentrations in the brain because the CNS tissue is protected by the BBB. The ability of compounds to cross the BBB can be predicted *in silico* using the SwissADME program.^28^ LCTB21 was recently discovered to be a potent inhibitor of hDyrk1A (IC_50_:2.4 nM), and an inactive isomer, iso-Leucettinib-21 (iso-LCTB21, IC_50_: > 10 μM), is available as an ideal negative control.^17,18^ Therefore, we inserted the structures of LCTB21 and iso-LCTB21 into SwissADME and analyzed their predicted brain penetrance properties using the “Brain Or IntestinaL EstimateD permeation method” (BOILED-Egg) (Figure 5A,B). This program visualizes the predicted ability of compounds to be absorbed by the human gastrointestinal tract (“egg white ”) and to pass through the BBB into the brain (“egg yellow ”) based on their polarity and lipophilicity.^29^ Both compounds were predicted to be well absorbed but exhibited only modest brain penetrance (Figure 5A,B).

**Figure 5:**
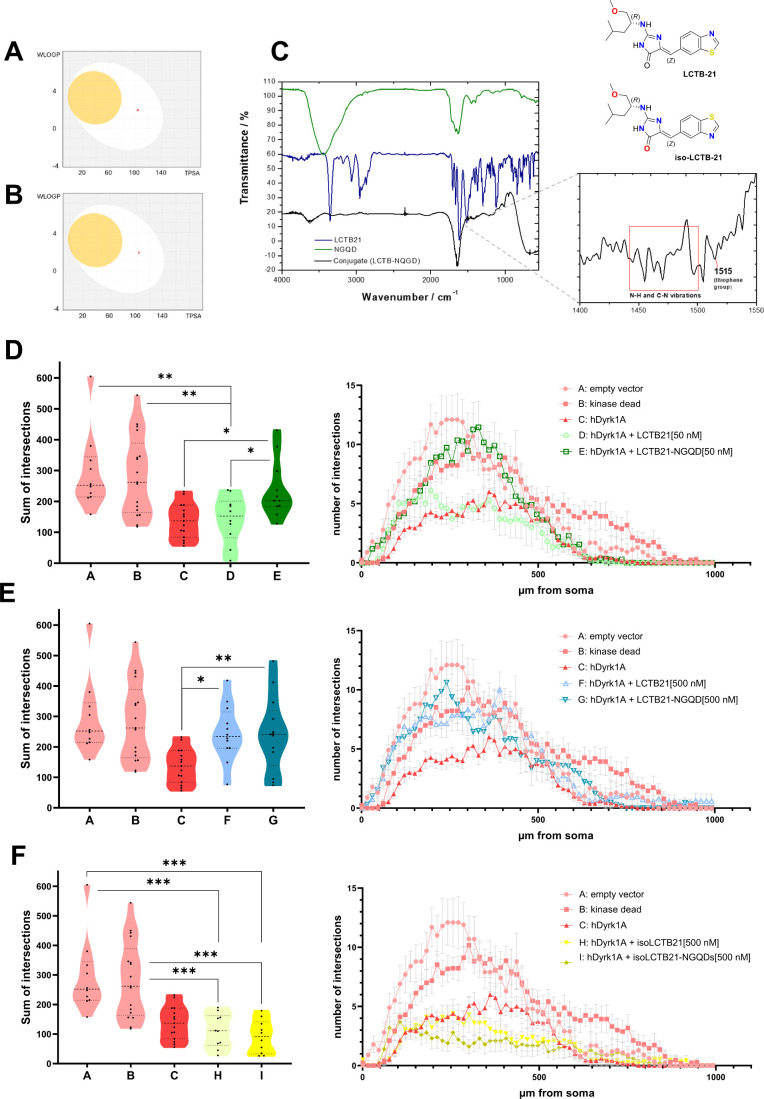
NGQDs support Leucettinib-21-mediated rescue of defects in PC dendrite morphology caused by hDyrk1A. SwissADME-derived boiled-egg visualization of the (A) active hDyrk1A kinase inhibitor Leucettinib-21 and (B) its inactive isomer iso-Leucettinib-21. Both compounds (red dots) were positioned in the egg-white, but not egg-yellow, suggesting only modest brain penetrance abilities. (C) FTIR spectrum of NGQDs (green), LCTB21 (blue) and the conjugates between NGQDs and Leucettinib-21 (black). The inset shows an enlargement of the region where LCTB21 vibrations appear in the conjugate spectrum. (D) Sholl analysis of dendritic structures of individual PCs with hDyrk1A overexpression treated with 50 nM LCTB21 alone (D, *n* = 10) or as an NGQD-conjugate (E, *n* = 11) by cardiac injection. The sum of the dendritic intersections is displayed on the left, and the number of intersections in relation to the distance of the dendritic structure from the PC soma is displayed on the right. (E) Sholl analysis of dendritic structures of individual PCs with hDyrk1A overexpression treated with 500 nM LCTB21 (F, *n* = 12) alone or as NGQD-conjugate (G, *n* = 13) by cardiac injection. The sum of the dendritic intersections is displayed on the right, and the number of intersections in relation to the distance of the dendritic structure from the PC soma is displayed on the left. Data from control groups A-B and hDyrk1A overexpression C are the same shown in D. (F) Sholl analysis of dendritic structures of individual PCs with hDyrk1A overexpression treated with 500 nM isoLCTB21 (H, *n* = 10) alone or as an NGQD-conjugate (I, *n* = 10) by cardiac injection. The sum of dendritic intersections is displayed on the right, the number of intersections in relation to the distance of the dendritic structure from the PC soma is displayed on the left. Data from control groups A-B and hDyrk1A overexpression C are the same shown in D.

As NGQDs possess excellent brain-penetrating properties, we incubated LCTB21 and iso-LCTB21 (10 μM each) with NGQDs (100 μg/mL) in water on a rotator (300 rpm) at room temperature for 16 h, followed by sterile filtration (0.22 μm). FTIR spectroscopy was used as a qualitative approach to investigate the possible interactions between the NGQD and the inhibitor. The FTIR spectrum (Figure 4C) revealed that the NGQD/LCTB21 mixture generated a spectrum (black) that represented a combination of the spectra generated by NGQDs (green) and LCTB21 (blue). Although the spectrum was dominated by the NGQD band due to their excess in the formulation, there were notably prominent peaks between 1711 and 1604 cm^–1^ associated with C = O vibrations in the substituted amides of LCTB21, which were more intense in the NGQD/LCTB21 mixture at 1671 cm^–1^, indicating the interaction of LCTB21 and its isomer with the nanoparticles. Other vibrational signals of the C = O, thiophene, C–N, and N–H groups of LCTB21 were shifted to longer wavelengths in the NGQD/LCTB21 conjugate, which, owing to the high concentration of NGQDs, could only be observed when the spectrum was stretched (see the inset in Figure 5C). Graphene-based nanomaterials interact with aromatic molecules through noncovalent mechanisms, including π–π stacking, hydrophobic interactions, and hydrogen bonding. These mechanisms enable the adsorption of therapeutic molecules onto the nanomaterial surface. Several studies have demonstrated the successful loading of drugs onto graphene quantum dots via these interactions.^53,74,75^ These spectroscopic changes are consistent with the adsorption of LCTB21 onto the surface or layers of the NGQDs, supporting the use of these nanoparticles as fluorescent drug-delivery systems.

### Conjugates of NGQDs and Leucettinib-21 Enhance the Rescue of Defects in PC Morphology Caused by hDyrk1A Overexpression

To address the potential of LCTB21 to rescue defects in PC morphology caused by hDyrk1A overexpression, zebrafish embryos were microinjected with PC-specific expression constructs containing hDyrk1A or hDyrk1A-KD at the one-cell stage (Figure 4A). LCTB21 or its inactive isomer iso-LCTB21 was delivered to these larvae with fluorescence in the cerebellum by cardiac microangiography at 3dpf either alone or as NGQD conjugates at different concentrations. The following day, zebrafish larvae containing individual green fluorescent PCs were selected and PC morphology analysis was performed using *in vivo* confocal microscopy recordings for PC reconstruction, followed by Sholl analysis to determine the dendritic complexity of individual PCs.

In PCs overexpressing hDyrk1A, the sum of dendritic intersections was significantly reduced compared to that in PCs not expressing hDyrk1A or expressing a kinase-inactive hDyrk1A-KD, but no significant difference in the sum of intersections was observed in hDyrk1A overexpressing PCs without compound treatment or treatment with 50 nM LCTB21 (Figure 5D). The number of intersections in relation to their distance from the PC soma revealed similar maximal numbers of approximately 6.0 (at a distance of 361 μm) intersections on average compared to approximately 12.1 (empty vector, 241–271 μm distance) and 10.2 (kinase dead, 301 μm distance) intersections, respectively, as maximal average values in controls. These findings showed that LCTB21 at a concentration of 50 nM is not sufficient to rescue dendritic defects caused by hDyrk1A overexpression.

Strikingly, when 50 nM LCTB21 was provided as an NGQD conjugate, the sum of intersections of PC dendrites increased significantly in the Sholl analysis and was raised to levels with no significant differences compared to the control PCs. Similarly, the values for the maximal number of intersections on average were reached at a distance of 301 μm from the PC soma and amounted to approximately 11.5 on average, as in the control PCs. This demonstrates that NGQDs strongly enhance the rescue properties of LCTB21 in inhibiting hDyrk1A kinase activity, likely by facilitating its entry into the brain.

At 3dpf an individual zebrafish larva weighed approximately 0.23 mg (n = 89) on average. Therefore, 22 nL injection volume of a 50 nM LCTB21 (MW: 358.46 g/mol) solution corresponds to an approximate LCTB21 dose of 1.71 ng/g body weight as concentration able to rescue altered PC morphology. This demonstrates the high *in vivo* affinity of LCTB21 for inhibiting Dyrk1A kinase activity.

However, all types of hDyrk1A expressing PCs displayed a shortened dendritic field independent of LCTB21, and NGQD/LCTB21 treatment terminated approximately 650 μm away from the soma, indicating that the rescue of dendritic structures with the NGQD-LCTB21 conjugates at 50 nM LCTB21 (termination at 631 μm, compared to 796 μm in empty vector and 856 μm in kinase-dead controls) was not complete (Suppl. Figure S2A).

When the concentration of LCTB21 was increased by 10-fold to 500 nM, the dendritic structures of hDyrk1A overexpressing PCs could be rescued by both the sum of intersections and the number of intersections per distance, peaking at approximately 391 μm from the PC soma (Figure 5E). Furthermore, no overall significant differences in the number of intersections in relation to the distance of the dendritic structures to the PC somata were observed between the control PCs and LCTB21 (10.0, at 391 μm distance) or NGQD/LCTB21 (10.6, at 241 μm distance)-treated specimens (Suppl. Figure S2B). These findings showed that, at this higher concentration, LCTB21 was able to fully rescue dendritic defects caused by hDyrk1A overexpression in PCs and did not require the adjuvant effect of NGQDs.

To confirm that the observed improvements in dendritic complexity of hDyrk1A expressing PCs were dependent on LCTB21 mediated inhibition of the hDyrk1A kinase, treatment with inactive iso-LCTB21 at a concentration of 500 nM was performed. Neither alone (4.4 intersections, at 256 μm) nor as an NGQD conjugate (3.7 intersections at 106 μm distance) was this isomer able to rescue the significant dendrite impairments of hDyrk1A expressing PCs, which were indistinguishable in the Sholl analysis of PCs expressing hDyrk1A without LCTB21 treatment (Figure 5F and Suppl. Figure S2C).

These findings indicate that LCTB21 can rescue the structural deficits in PCs caused by Dyrk1A overexpression in a concentration-dependent manner. However, the effective concentration of LCTB21 was significantly reduced in the presence of NGQDs, likely because of the ability of these nanoparticles to carry LCTB21 into the CNS. It is likely that NGQDs exert their rescue-enhancing effect by easily crossing the BBB, thereby facilitating the entry of conjugated LCTB21 into the CNS and increasing the effective LCTB21 concentration in the brain. This allows for a reduction in the applied dose for rescuing Dyrk1A overexpressing neurons and reduces the amount of LCTB21 in tissues other than the brain that are not targeted by the LCTB21-application, thereby significantly reducing potentially detrimental side effects.

## Conclusions

We developed a fluorescent nanomaterial with promising optical and electronic properties, combined with a sufficiently small size to enable translocation across the blood–brain barrier, which represents one of the major challenges in the treatment of central nervous system diseases. In addition to its use as a fluorescent probe, this nanomaterial was demonstrated to enter the brain in zebrafish larvae and act as a potentially effective carrier for the delivery of therapeutic inhibitors targeting diseases such as DS with overexpression of Dyrk1A, highlighting the dual functionality of the synthesized NGQDs for imaging and drug delivery applications. Currently, neither the mode of BBB passage nor the mechanisms by which NGQDs conjugated to LCTB21 rescue dendrite morphogenesis of Dyrk1A overexpressing PCs are fully understood. Based on the ability of NGQDs to mark cellular membranes in combination with their negative charge, the amphipathic nature of NGQDs is likely to facilitate their passage across cellular barriers. Whether NGQDs carry LCTB21 across the BBB in a conjugated state or indirectly facilitate the entry of LCTB21 into the brain remains unclear. Nevertheless, because the inactive isomer iso-LCTB21 incubated with NGQDs was unable to rescue the dendrite morphology defects of Dyrk1A overexpressing PCs, the NGQDs themselves did not exert a rescue effect. Because LCTB21 at higher concentrations can rescue the observed PC phenotypes alone, the most likely explanation is that NGQDs either increase the effective concentration of this compound in the brain or promote its rescue efficacy by enhancing the affinity of LCTB21 to bind to Dyrk1A. It cannot be excluded that LCTB21 rescues PC dendrite morphology in Dyrk1A overexpressing PCs indirectly by inhibiting other related kinases, such as CLK4, or other nonkinase targets. Given the high affinity of LCTB21 for specifically inhibiting Dyrk1A at subnanomolar concentrations,^17,18^ we consider this possibility unlikely.

To further apply NGQDs for therapeutic approaches, it is important to address the clearance of these nanoparticles. We observed a rapid exit of NGQDs from the vasculature and dispersion of NGQDs into the surrounding tissues. Therefore, the decrease in fluorescence within a few hours after the injection of NGQDs into the bloodstream of zebrafish larvae is likely due to the dilution effects of these nanoparticles. Semiconductor quantum dots have been reported to accumulate in the lymphatic system of zebrafish larvae^9,40,76^ from where they are likely removed via the venous and nephric systems. Whether this excretion route also applies to NGQDs or whether NGQDs are metabolized by cells after uptake remains an exciting area of research.

Notably, the potential of NGQDs is not limited to their role as efficient BBB-penetrating agents and drug carriers because they possess therapeutic potential. For example, GQDs have been demonstrated to inhibit Aβ peptide aggregation and amyloid plaque formation, which are characteristic of AD.^6^ In addition, GQDs diminished the aggregation of α-Synuclein and TDP-43 in mouse models of PD and amyotrophic lateral sclerosis.^77,78^ Combining these aggregation-inhibiting properties with the administration of therapeutic compounds has already been successfully demonstrated, using GQDs conjugated to aggregation-inhibiting compounds or neuroprotective peptides to rescue neurological deficits in mouse models of AD.^7,8,79,80^ Therefore, LCTB21’s synergy with NGQDs is a promising approach for mitigating neurodegeneration and enhancing neuronal health. Because the BBB of zebrafish and mice is evolutionarily well conserved,^81^ testing the passage of NGQDs across the BBB in mammals will be a worthwhile approach in this direction.

## Supplementary Material



## Data Availability

The data are presented in the article. The data presented in this study are available upon request from the corresponding authors.

## Notes

Time-dependent zeta potential measurements of N-GQD aqueous solutions over a 60-day period, evaluating colloidal stability (Figure S1); statistical significance values associated with Sholl analysis used to quantify dendritic complexity (Figure S2) (PDF)
