# Supplementary material for: Nitrogen-Doped Graphene Quantum Dots Conjugated to Leucettinib-21 Rescue Differentiating Zebrafish Purkinje Cells by Inhibiting Dyrk1A Kinase
Source: ACS Appl Nano Mater. 2026 Apr 20;9(18):8023–38. doi: 10.1021/acsanm.6c00239 (PMC13162200; doi:10.1021/acsanm.6c00239)
Supplement: Supplementary file 1 [file an6c00239_si_001.pdf]

## Supporting Information

# Nitrogen-Doped Graphene Quantum Dots Conjugated to Leucettinib-21 Rescue Differentiating Zebrafish Purkinje Cells by Inhibiting Dyrk1A Kinase

Luiza Araújo Gusmão[1,2,§,*], Annemarie Metzke[1,§], Emmanuel Deau[3], Laurent Meijer[3], Antonio Claudio Tedesco[2], Reinhard W. Köster[1,*]

[1] Division of Cellular and Molecular Neurobiology, Zoological Institute, Technische Universität Braunschweig, Spielmannstraße 7, 38106 Braunschweig, Germany

[2] Department of Chemistry, Center of Nanotechnology and Tissue Engineering, Photobiology and Photomedicine Research Group, Faculty of Philosophy, Sciences and Letters of Ribeirão Preto, University of São Paulo, São Paulo, Brazil.

[3] Perha Pharmaceuticals, Perharidy Research Center, 29680 Roscoff, France

*Correspondence to:* luizaaraujo@usp.br, r.koester@tu-braunschweig.de

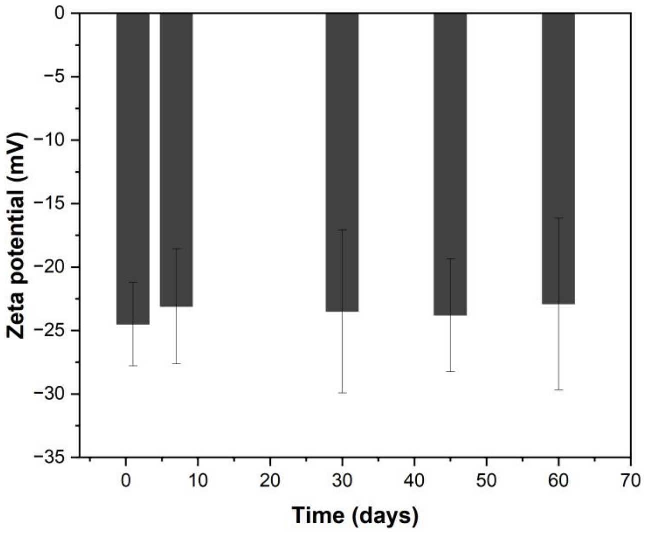


**Figure S1:** Monitoring of the zeta potential of N-GQD solution in water over a period of 60 days. Each measurement was taken in triplicate, and the results are presented as mean and standard deviation.

A

| | | |
|---|---|---|
| empty vector vs. 50 nM | **** | <0,0001 |
| empty vector vs. 50 Mix | *** | 0,0007 |
| kinase dead vs. 50 nM | **** | <0,0001 |
| kinase dead vs. 50 Mix | *** | 0,0001 |
| hDyrk1A vs. 50 nM | ns | >0,9999 |
| hDyrk1A vs. 50 Mix | * | 0,0237 |
| 50 nM vs. 50 Mix | ** | 0,0071 |

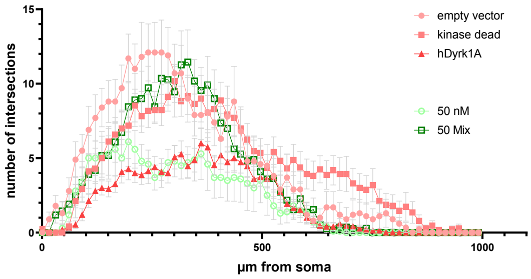


B

| | | |
|---|---|---|
| empty vector vs. 500 nM | ns | >0,9999 |
| empty vector vs. 500 Mix | ns | >0,9999 |
| kinase dead vs. 500 nM | ns | >0,9999 |
| kinase dead vs. 500 Mix | ns | 0,478 |
| hDyrk1A vs. 500 nM | **** | <0,0001 |
| hDyrk1A vs. 500 Mix | **** | <0,0001 |
| 500 nM vs. 500 Mix | ns | 0,5694 |

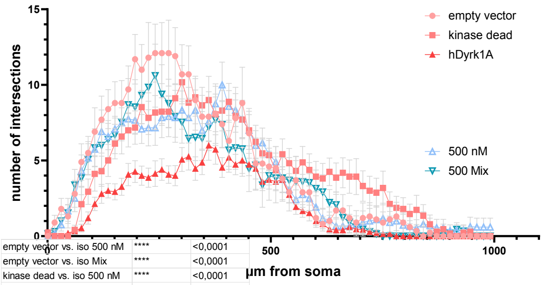


| | | |
|---|---|---|
| empty vector vs. iso 500 nM | **** | <0,0001 |
| empty vector vs. iso Mix | **** | <0,0001 |
| kinase dead vs. iso 500 nM | **** | <0,0001 |
| kinase dead vs. iso Mix | **** | <0,0001 |
| hDyrk1A vs. iso 500 nM | ns | >0,9999 |
| hDyrk1A vs. iso Mix | ns | >0,9999 |
| iso 500 nM vs. iso Mix | ns | >0,9999 |

C

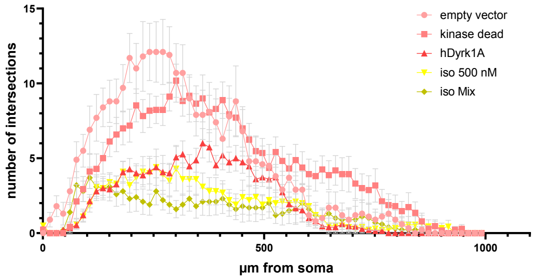


**Figure S2.** NGQDs support Leucettinib-21-mediated rescue of defects in PC dendrite morphology caused by hDyrk1A. **(A)** Significance values for Sholl analysis of dendritic structure intersections with concentric circles in relation to the distance of the dendritic structure from the PC soma (50 nM LCTB21, see Fig. 5D). **(B)** Significance values for Sholl analysis of dendritic structure intersections with concentric circles in relation to the distance of the dendritic structure from the PC soma (500 nM LCTB21, see Fig. 5E). **(C)** Significance values for Sholl analysis of dendritic structure intersections with concentric circles in relation to the distance of the dendritic structure from the PC soma (500 nM iso-LCTB21, see Fig. 5F).
